# Integrated Metagenomic and Untargeted Metabolomic Analyses Characterize Enramycin-Associated Colonic Microbial and Metabolic Changes in Weaned Rabbits

**DOI:** 10.3390/ani16172764

**Published:** 2026-09-03

**Authors:** Xiaowen Luo, Jingzhe Zhang, Qiman Yang, Yongzhen Sun, Yangyang Zhao, Xueting Hu, Xinyu Wang, Lang Hu, Hongmei Xu, Lin Huang, Min Lei, Congyan Li, Liangde Kuang, Wei Fu

**Affiliations:** 1 College of Animal & Veterinary Sciences, Southwest Minzu University, Chengdu 610041, China; 2 Key Laboratory of Qinghai-Tibetan Plateau Animal Genetic Resource Reservation and Utilization, Ministry of Education, Southwest Minzu University, Chengdu 610041, China; 3 Animal Genetic Breeding and Reproduction Key Laboratory of Sichuan Province, Sichuan Animal Science Academy, Chengdu 610066, China

**Keywords:** enramycin, weaned rabbits, colonic microbiota, untargeted metabolomics, antimicrobial stewardship

## Abstract

Enramycin is used as a dietary antimicrobial in young animals, but its effects on rabbit colonic ecology remain insufficiently characterized. This study evaluated the effects of enramycin in 60 weaned rabbits randomly assigned to a control or enramycin-treated group (60 mg/kg) for 14 days (*n* = 30/group). Clinical outcomes were evaluated in all rabbits, whereas paired colonic multi-omics profiling was performed in six randomly selected survivors per group with no recorded diarrhea. Enramycin reduced diarrhea incidence without affecting body-weight development, organ indices, or mortality. Metagenomic analysis revealed unchanged alpha diversity but significant alterations in microbial community structure, including decreased *Verrucomicrobiota*, *Akkermansia*, and *Alistipes* and increased Cyanobacteria and *Avigastranaerophilus*. Untargeted metabolomics identified 332 features meeting the predefined screening criteria, including 143 upregulated and 189 downregulated metabolites. Prominent pathway annotations included phenylpropanoid biosynthesis and arachidonic acid metabolism. Resistome analysis demonstrated that treatment drove shifts in streptothricin-, aminoglycoside- and glycopeptide-related resistance signatures. The integrated analysis identified associations between four selected genera and the top 30 differential metabolites. Overall, dietary enramycin was associated with a short-term reduction in diarrhea incidence and concurrent changes in selected colonic microbial, metabolic, and resistance gene features. These findings extend enramycin evaluation beyond conventional clinical outcomes and highlight the importance of considering broader ecological effects when assessing dietary antimicrobial exposure in weaned rabbits.

## Simple Summary

Digestive disorders are common after weaning and can compromise rabbit health. Enramycin is a dietary antimicrobial used in young animals, but its broader effects on the intestinal ecosystem remain unclear. This study evaluated the short-term effects of dietary enramycin supplementation in weaned rabbits by combining clinical observations with colonic microbiota and metabolite analyses. During the 14-day trial, enramycin significantly reduced diarrhea incidence but did not improve body-weight development or major organ indices. Supplementation was accompanied by alterations in selected microbial groups, metabolic features, and antibiotic resistance-related characteristics. However, because these ecological analyses were conducted in a limited subset of surviving rabbits without recorded diarrhea, these changes should not be interpreted as the direct mechanism underlying the reduced diarrhea incidence. Nevertheless, the findings indicate that enramycin may provide short-term benefits for diarrhea control while simultaneously influencing the intestinal ecological environment. Therefore, enramycin dosage and duration should be carefully considered in weaned rabbits. The results highlight the importance of weighing potential short-term benefits against broader ecological effects when evaluating dietary antimicrobial use.

## 1. Introduction

Digestive disorders during the post-weaning period remain a major challenge in meat rabbit production and health management [1]. At this developmental stage, digestive function and the intestinal microbiota are still maturing, making weaned rabbits particularly susceptible to dietary, environmental, and pharmacological disturbances [2,3,4]. Dietary antimicrobials have been used to mitigate intestinal health problems in young animals, but their effects cannot be evaluated solely from diarrhea incidence or body-weight outcomes [5]. Antimicrobial exposure may also alter microbial community composition, metabolic activity, and the distribution of resistance genes within the intestinal ecosystem [6,7]. Therefore, a more comprehensive assessment linking health-related outcomes with intestinal ecological responses is needed.

Enramycin is a polypeptide antibiotic produced by *Streptomyces fungicidicus* that predominantly acts against Gram-positive bacteria by inhibiting peptidoglycan synthesis and is minimally absorbed from the intestine [8,9,10]. Enramycin has been investigated as a dietary antimicrobial in piglets [11], broilers [12], and rabbits [13]. However, previous rabbit studies have primarily focused on production traits [4], nutrient utilization [14], and diarrhea-related outcomes [15]. Whether short-term dietary enramycin exposure is accompanied by changes in the colonic microbial community, metabolic environment, and resistance gene profiles remains insufficiently characterized [16,17,18]. Its limited intestinal absorption further suggests that local ecological responses may not be adequately reflected by conventional health and body-weight measurements. This gap is particularly relevant for therapeutic application because short-term improvement in diarrhea-related outcomes may occur with changes in microbial functional potential [17,18], metabolite exchange [17], and resistance gene profiles [7].

Metagenomics and untargeted metabolomics provide complementary approaches for characterizing intestinal ecological responses to antimicrobial exposure [19]. Metagenomics describes microbial composition, functional potential, and resistance gene profiles [20,21], whereas untargeted metabolomics captures broad changes in small-molecule profiles associated with microbial activity and host–microbiota interactions [22,23]. Integrating these approaches using matched colonic samples can characterize concurrent microbial and metabolic responses and identify statistical associations between the two datasets [24,25]. Integrating these approaches can provide a systems-level perspective on intestinal ecological responses and facilitate a more comprehensive evaluation of antimicrobial interventions in rabbit production. Such integration is useful for distinguishing direct production-related responses from broader remodeling of the intestinal microenvironment, especially when clinical outcomes and growth indices are not fully consistent.

We hypothesized that short-term dietary enramycin supplementation would reduce diarrhea-related outcomes while altering selected features of the colonic ecosystem. Accordingly, the present study integrated clinical and body-weight measurements with colonic metagenomic, untargeted metabolomic, and resistome profiling in weaned rabbits. The objectives were to characterize enramycin-associated microbial, metabolic, and resistance gene changes and to examine associations between selected microbial genera and differential metabolites. This integrated framework was intended to extend the evaluation of dietary enramycin beyond conventional health and body-weight outcomes and to provide an ecological basis for subsequent dose–response, withdrawal, and alternative-intervention studies.

## 2. Materials and Methods

### 2.1. Experimental Design

All experimental procedures were approved by the Academic Ethics Committee of Southwest Minzu University (approval no. SMU-202309004) and were conducted in accordance with institutional guidelines for the care and use of experimental animals. Shuxing No. 1 meat rabbits were obtained from the meat rabbit research base of the Sichuan Academy of Animal Science. Sixty healthy weaned rabbits (35 days of age) with comparable body weights and an equal male-to-female ratio were individually housed and randomly allocated to a control (CN) or enramycin-supplemented (AN) group, with 30 animals per group. Each rabbit was housed in a separate cage and served as an independent experimental unit. Both groups were maintained in the same facility at 19–25 °C and 60–65% relative humidity [26,27]. Feeding, ventilation, routine cage cleaning, and disinfection were applied consistently to both groups. Personnel preparing and providing the diets were aware of treatment allocation, whereas fecal assessment was performed by personnel unaware of the allocation. The CN group received the basal diet, whereas the AN group received the same diet supplemented with 0.75 g/kg enramycin premix (8% active content). The trial lasted 14 consecutive days under a natural photoperiod, with ad libitum access to feed and water. At the end of the trial, six rabbits per group (three males and three females) were randomly selected [17,18,28] within sex strata for terminal sampling and matched omics analyses. Selection was based on treatment group and sex balance and not on health status, diarrhea history, or any omics result. Review of the clinical records after selection confirmed that none of the 12 selected rabbits had a recorded diarrhea episode. This sampling strategy balanced animal welfare, analytical feasibility, and sex representation according to the 3R principles of replacement, reduction, and refinement. All selected samples were retained in the final analyses. The selected rabbits were used to assess growth and development indices, colonic microbiota and metabolomic profiles, and the integrated analysis of differential bacterial genera and core differential metabolites. No a priori power calculation was performed for the omics subset; the use of six biological samples per group was comparable with recent rabbit multi-omics studies using six samples per condition [17,18].

### 2.2. Experimental Materials

The basal diet was supplied by Sichuan Xinye Meilin Co., Ltd. (Nanchong, China), as Jiajie Grade II complete feed for meat rabbits. It contained grade I corn, soybean meal (43%), grade II wheat, wheat bran, alfalfa pellets, dicalcium phosphate, limestone powder (38% Ca), sodium chloride, L-lysine hydrochloride (98.5%), vitamins A, D, and E, basic copper chloride (50% Cu), manganese sulfate (31.8% Mn), ferrous sulfate heptahydrate, and zinc sulfate (34.5% Zn), consistent with the commercial feed description reported in previous studies using Shuxing No. 1 rabbits [26,27]. According to the manufacturer’s label, the declared nutrient specifications were crude protein ≥ 14%, crude fat 2%, crude fiber 10–20%, crude ash ≤ 12%, calcium 0.5–1.0%, a calcium-to-phosphorus ratio of 2:1, sodium chloride 0.3–0.8%, moisture ≤ 12.5%, and lysine ≥ 0.9%.

Enramycin was purchased from Shandong Shengli Bioengineering Co., Ltd. (Jining, China), as an 8% premix containing 8 g of enramycin per 100 g of product. The AN diet contained 0.75 g premix/kg diet; thus, 0.75 g/kg × 0.08 = 0.060 g/kg, corresponding to 60 mg enramycin/kg diet. The premix was blended stepwise with a small quantity of basal diet, mixed thoroughly with the remaining feed, and then pelleted for uniform distribution. Both groups received the same basal diet. No separate carrier was added to the CN diet, and the product met the quality-control requirements for veterinary drugs.

Enramycin is a macromolecular cyclic peptide antibiotic that mainly targets cell-wall synthesis in Gram-positive bacteria and is minimally absorbed in the intestine. Previous studies have reported that 5–20 mg/kg enramycin improves growth performance in weaned piglets, while 80 mg/kg remains within the safe range [11]. In addition, Guo et al. reported no obvious adverse effects when 200 mg/kg enramycin was included in the diet of growing rabbits [13]. Ngoh et al. also reported that dietary supplementation with 100 mg/kg enramycin was well tolerated in broiler chickens [12]. Based on these previous studies, enramycin was supplemented at a final dietary concentration of 60 mg/kg in the present experiment. This dosage was selected within the safe range reported in previous animal studies.

### 2.3. Sample Collection

At the end of the feeding trial, the six rabbits selected from each group, as described in Section 2.1, comprised three males and three females. The rabbits were fasted for 12 h before terminal sampling. Terminal body weight was recorded for all rabbits surviving to day 14, whereas the thymus, spleen, kidneys, liver, middle-colon contents, and colon-wall tissue were collected from the six terminally sampled rabbits per group. The collected colonic samples were immediately frozen in liquid nitrogen and stored at −80 °C.

The colon was selected as the target intestinal segment because it plays a critical role in diarrhea-associated intestinal dysfunction and microbial fermentation processes [18,29]. Colonic microbiota dysbiosis is a key factor in the occurrence and development of intestinal diseases in rabbits [18], and the metabolomic profile of colonic contents is closely related to intestinal functional status. Previous studies have shown that colonic metabolomics can be used to reveal the mechanisms underlying intestinal dysfunction [30]. In addition, compared with cecal contents, colonic contents contain higher levels of terminal fermentation products, such as short-chain fatty acids, and therefore better reflect the terminal metabolic state of the intestinal microecosystem [4]. For these reasons, the colon was selected for analysis in this study rather than the cecum. The use of matched mid-colonic contents for metagenomic and untargeted metabolomic analyses ensured that both datasets represented the same anatomical site and biological samples. This sampling design was intended to characterize colonic microbial and metabolic features and should not be considered a substitute for analysis of the cecal microbiota or the entire hindgut ecosystem.

### 2.4. Measurement of Body-Weight Development, Organ Indices, and Diarrhea Incidence

Mental status and fecal characteristics were assessed once daily in all rabbits throughout the 14-day trial. A multilevel fecal-scoring system was not used; instead, a predefined binary classification was applied. Observers had routine experience in rabbit health monitoring, received instructions in the predefined criteria, and were unaware of treatment allocation. Unformed or runny feces and watery feces were classified as diarrhea [18,31]; soft but formed feces, normal feces, and other appearances not meeting these criteria were not classified as diarrhea. No minimum duration or number of consecutive days was required. A rabbit was classified as a diarrhea case when at least one qualifying episode was observed during the trial; each rabbit was counted only once, even when episodes occurred on multiple days. We calculated diarrhea incidence by dividing the number of rabbits with at least one recorded episode by the 30 rabbits initially allocated to each group. Initial body weight was recorded in all 30 rabbits per group. Terminal body weight was measured after a 12 h fast in rabbits surviving to day 14 (CN, *n* = 6; AN, *n* = 6), whereas organ weights and indices were obtained from the six terminally sampled rabbits per group. Body weight change and organ indices were calculated as follows:

Body weight change = pre-slaughter live weight − initial body weight;

Organ index = organ weight/terminal body weight × 100%.

### 2.5. Colonic Metagenomic Analysis

Total microbial DNA was extracted from 0.3 g of colonic contents using the cetyltrimethylammonium bromide (CTAB) method [32]. DNA integrity and purity were assessed using 1% agarose gel electrophoresis and a NanoDrop 2000 spectrophotometer (Amersham Biosciences Corp., Piscataway, NJ, USA), with qualified samples defined by OD260/280 values of 1.8–2.0 and OD260/230 values greater than 1.8. Qualified DNA was fragmented to approximately 450 bp using a Covaris M220 (Covaris LLC, Woburn, MA, USA) instrument, and paired-end libraries were prepared using the TruSeq DNA Sample Prep Kit (Omega Bio-tek, Norcross, GA, USA). Libraries were sequenced by LingEn Biotechnology Co., Ltd. (Shanghai, China) using Illumina paired-end sequencing (PE150), and base calling was used to generate FASTQ-format raw reads containing sequence and base-quality information. Per-sample raw- and clean-read statistics are provided in Tables S1 and S2.

Raw reads were processed using Trimmomatic (v0.36) with the parameters ILLUMINACLIP: adapters.fa:2:30:10, SLIDINGWINDOW:4:15, and MINLEN:75. Adapter sequences were removed, low-quality bases with Phred scores below 20 were trimmed from the 3′ end, and a read was discarded if the retained sequence still contained bases below Q10, more than 10% undetermined bases, or a length below 75 bp. Reads aligned to the rabbit host genome were removed before downstream analysis; the numbers and retention rates of non-host read pairs are reported in Table S3.

Host-filtered reads were assembled de novo using MEGAHIT (v1), and the per-sample assembly statistics are provided in Table S4. For sequence-location annotation, contigs of at least 1000 bp were analyzed using PlasFlow with a classification threshold of 0.9 and assigned as chromosomal, plasmid-derived, or unclassified sequences (Table S5).

Open reading frames were predicted from assembled sequences using MetaProdigal (v2.6.3), and the gene-prediction statistics are summarized in Table S6. Predicted gene sequences from all samples were clustered using CD-HIT at 95% sequence identity and 90% coverage, and the longest sequence in each cluster was retained to construct the non-redundant gene catalog. We quasi-mapped clean reads to the non-redundant catalog using Salmon and normalized gene abundance as transcripts per million (TPM): TPMi = [(Ni/Li)/Σj(Nj/Lj)] × 10^6^, where Ni is the number of reads assigned to gene i and Li is its sequence length. The resulting gene-abundance matrix is provided in Table S7.

Predicted protein sequences were compared with the NCBI non-redundant protein (NR) database using diamond 0.9.22.123 blastp --evalue 0.00001 --id 70, and taxonomic abundance at each rank was calculated by summing the TPM abundances of genes assigned to the corresponding taxon. Phylum- and genus-level abundance profiles are provided in Tables S8 and S9. Alpha diversity was assessed using the Simpson index. Bray–Curtis dissimilarities were calculated from the TPM-normalized microbial gene-abundance matrix, and principal co-ordinate analysis (PCoA) was used to visualize differences in overall abundance profiles between the CN and AN groups. PCoA1 and PCoA2 were displayed with 95% confidence ellipses and marginal density curves. Group differences were tested by permutational multivariate analysis of variance (PERMANOVA) using the same Bray–Curtis distance matrix and 999 permutations; the pseudo-F statistic, R^2^, and permutation *p* value were reported. Homogeneity of multivariate dispersion was evaluated by permutational analysis of multivariate dispersions (PERMDISP) using the betadisper and permutest functions in the vegan package in R, with distances calculated to the group spatial median and 999 permutations. Genus-level differences were evaluated using the Wilcoxon rank-sum test, and the corresponding nominal *p* values and Benjamini–Hochberg-adjusted false discovery rate (FDR) values are reported in Table S14. Microbial genes were annotated against the Kyoto Encyclopedia of Genes and Genomes (KEGG) using KofamScan and profile hidden Markov models to describe functional composition. Antibiotic resistance genes were annotated against the Structured Antibiotic Resistance Gene Reference Database (SARG) v2.3 [7], and abundance profiles were summarized at the individual gene and resistance-mechanism levels. The functional and resistance gene annotation outputs are provided in Tables S10 and S11.

### 2.6. Untargeted Metabolomic Analysis of Colonic Contents

Colonic contents (0.1 g) were extracted with 500 μL of 80% methanol–water. Samples were vortexed, incubated on ice for 5 min, and centrifuged at 15,000× *g* and 4 °C for 20 min. The supernatant was diluted with mass-spectrometry-grade water to a final methanol concentration of 53% and centrifuged again under the same conditions. Equal-volume aliquots from all samples were pooled as quality-control samples. Three quality-control injections were performed at the beginning of the sequence, followed by one quality-control injection after every 10 analytical samples; 53% methanol–water served as the blank. Chromatographic separation was performed using a Vanquish ultra-high-performance liquid chromatography system coupled to an Orbitrap Q Exactive high-resolution mass spectrometer. A Hypersil Gold C18 column was operated at 40 °C and 0.2 mL/min. In positive-ion mode, mobile phases A and B were 0.1% formic acid in water and methanol, respectively; in negative-ion mode, mobile phase A was 5 mM ammonium acetate at pH 9.0 and mobile phase B was methanol. The scan range was *m*/*z* 100–1500. The electrospray-ionization settings were a spray voltage of 3.5 kV, sheath-gas pressure of 35 psi, auxiliary-gas flow of 10 L/min, capillary temperature of 320 °C, S-lens radio-frequency level of 60, and auxiliary-gas heater temperature of 350 °C. Data-dependent tandem mass spectrometry (MS/MS) acquisition was performed in positive- and negative-ion modes.

Raw mass-spectrometry files were processed using Compound Discoverer 3.1 (Thermo Fisher Scientific, Waltham, MA, USA) by Shanghai LingEn Biotechnology Co., Ltd. Peak alignment used a retention-time tolerance of 0.2 min and mass tolerance of 5 ppm; peak extraction used a mass tolerance of 5 ppm, intensity tolerance of 30%, signal-to-noise threshold of 3, and the adduct-ion and minimum-intensity settings in the company workflow. Peak extraction, alignment, integration, blank-background removal, and total peak-area normalization were performed, and features with a coefficient of variation below 30% in quality-control samples were retained. Molecular formulae were predicted from precursor *m*/*z*, mass error, and adduct information. Features with liquid chromatography–tandem mass spectrometry (LC-MS/MS) spectra were matched to fragment-ion and collision-energy information in the mzCloud and mzVault high-resolution spectral libraries, whereas MassList was used for accurate-mass matching. Chemical classes were assigned using the ClassyFire/ChemOnt hierarchy, and KEGG, HMDB, and LIPID MAPS identifiers were recorded when available. No metabolites were confirmed by comparison with authentic standards analyzed under the same conditions. In accordance with Metabolomics Standards Initiative terminology, mzCloud/mzVault spectral matches were treated as putative level-2 annotations and accurate-mass-only MassList matches as level-3 annotations [33]. Because a per-feature annotation-source field was not retained in Table S12, all metabolite names are conservatively reported as putatively annotated. The available annotation metadata in Table S12 include retention time, measured *m*/*z*, molecular formula, molecular weight, database identifiers, chemical classification, group means, log2 fold-change, nominal *p* value, FDR value, and VIP value.

The normalized metabolite-abundance matrices generated in positive- and negative-ion modes were merged for between-group multivariate analysis in MetaboAnalystR 2.0. Data were log-transformed and Pareto scaled before analysis. We first used unsupervised principal component analysis (PCA) to summarize overall sample variation, visualize within- and between-group distributions, and identify potential outliers; the score plot included 95% confidence ellipses. We then used orthogonal partial least-squares discriminant analysis (OPLS-DA) to separate variation associated with group classification from variation orthogonal to that classification. Model performance was summarized using R^2^X, R^2^Y, and Q^2^ and evaluated by sevenfold cross-validation and a 1000-permutation test. An S-plot was generated using the covariance p[1] and correlation p(corr)[1] of each feature with the predictive component. Differential metabolites were screened using a fold-change of ≥1.2 or ≤1/1.2, a variable importance in projection (VIP) value of ≥1, and an independent-samples *t*-test *p* value of ≤0.05. Feature-level *p* values were also adjusted using the Benjamini–Hochberg procedure and are reported as FDR values in Table S12; the original 332-feature set was defined by the predefined fold-change, VIP, and nominal *p* value criteria. The complete metabolite table includes molecular formula, molecular weight, retention time, *m*/*z*, database identifiers, chemical classification, group means, log2 fold-change, *p* value, FDR value, and VIP value (Table S12). Differential metabolites were mapped to the Kyoto Encyclopedia of Genes and Genomes for pathway annotation and enrichment analysis [34]. The corresponding pathway-level *p* and q values are provided in Table S13.

### 2.7. Microbiota–Metabolite Association Analysis

Four target genera (*Akkermansia*, *Alistipes*, *Avigastranaerophilus*, and *CAG-873*) were selected from the genus-level group-comparison table, and the top 30 most significant differential metabolites with the lowest nominal *p* values were selected from Table S12. Genus- and metabolite-abundance matrices were constructed for the matched samples, and abundance values were log10-transformed before association analysis.

Euclidean distance matrices were constructed from the log10-transformed genus and metabolite profiles. For each genus–metabolite combination, Mantel tests using Spearman correlation and 9999 permutations were used to evaluate association strength. Pearson correlation analysis was performed among the top 30 metabolites to characterize metabolite covariation patterns. *p* values from the original metabolite group comparisons, Pearson correlation tests, and Mantel tests were adjusted within their respective test families using the Benjamini–Hochberg procedure. Only Mantel links with FDR < 0.05 were retained for visualization.

The triangular heatmap displayed Pearson correlation coefficients among the top 30 metabolites, with asterisks indicating FDR-adjusted significance. The four target genera were displayed as nodes to the right of the heatmap. Link color represented the Mantel FDR category, link width represented the magnitude of Mantel’s *r*, and line type represented positive or negative association direction. Colored points above the heatmap indicated the Class I chemical category of each metabolite. Figures were generated in R (v4.2.3) using the ggnewscale and ggplot2 packages. All FDR-retained Mantel links displayed in the present network were positive. This association analysis describes statistical relationships and does not establish causal microbiota–metabolite interactions.

### 2.8. Statistical Analysis

Statistical analyses were performed using SPSS 22.0 and GraphPad Prism 9. Each individually housed rabbit was treated as an independent experimental unit. Before parametric analysis, continuous data were assessed for normality and homogeneity of variance, and potential outliers were examined. Independent-samples *t*-tests were used when the parametric assumptions were satisfied; appropriate non-parametric tests were used when those assumptions were not met. Continuous variables are presented as the mean ± standard deviation. Diarrhea incidence and mortality were analyzed using 2 × 2 contingency tables based on all 30 rabbits per group. Pearson’s chi-square test was used for diarrhea incidence because all expected cell counts were at least 5, whereas a two-sided Fisher’s exact test was used for mortality because expected cell counts were below 5. Relative risk (RR) comparing the AN group with the CN group and its 95% confidence interval were calculated using the Koopman asymptotic score method. Company-generated omics analyses followed the procedures described above: genus-level microbial comparisons used the Wilcoxon rank-sum test, whereas differential metabolites were screened using independent-samples *t*-tests together with the stated fold-change and VIP thresholds. Selected resistance gene and resistance-mechanism features were compared using independent-samples *t*-tests. Statistical significance was set at *p* < 0.05, with * indicating *p* < 0.05.

## 3. Results

### 3.1. Effects of Enramycin on Body-Weight Development, Diarrhea Incidence, and Organ Indices

No significant group differences were observed in initial body weight, terminal body weight, organ weights and indices in the terminally sampled subset (*n* = 6/group; *p* > 0.05; Table 1). In the full experimental population, diarrhea was recorded in 8/30 rabbits (26.67%) in the CN group and 2/30 rabbits (6.67%) in the AN group (χ^2^ = 4.320, *p* = 0.038; RR = 0.25, 95% CI: 0.062–0.944). Mortality was recorded in 4/30 rabbits (13.33%) in the CN group and 2/30 rabbits (6.67%) in the AN group and did not differ significantly (two-sided Fisher’s exact *p* = 0.671; RR = 0.50, 95% CI: 0.111–2.191). Thus, short-term dietary enramycin supplementation significantly reduced diarrhea incidence but did not improve body-weight development or organ indices during the 14-day trial.

### 3.2. Effects of Enramycin on the Structure and Function of the Colonic Microbiota in Weaned Rabbits

#### 3.2.1. Microbial Diversity Characteristics

**Figure 1:**
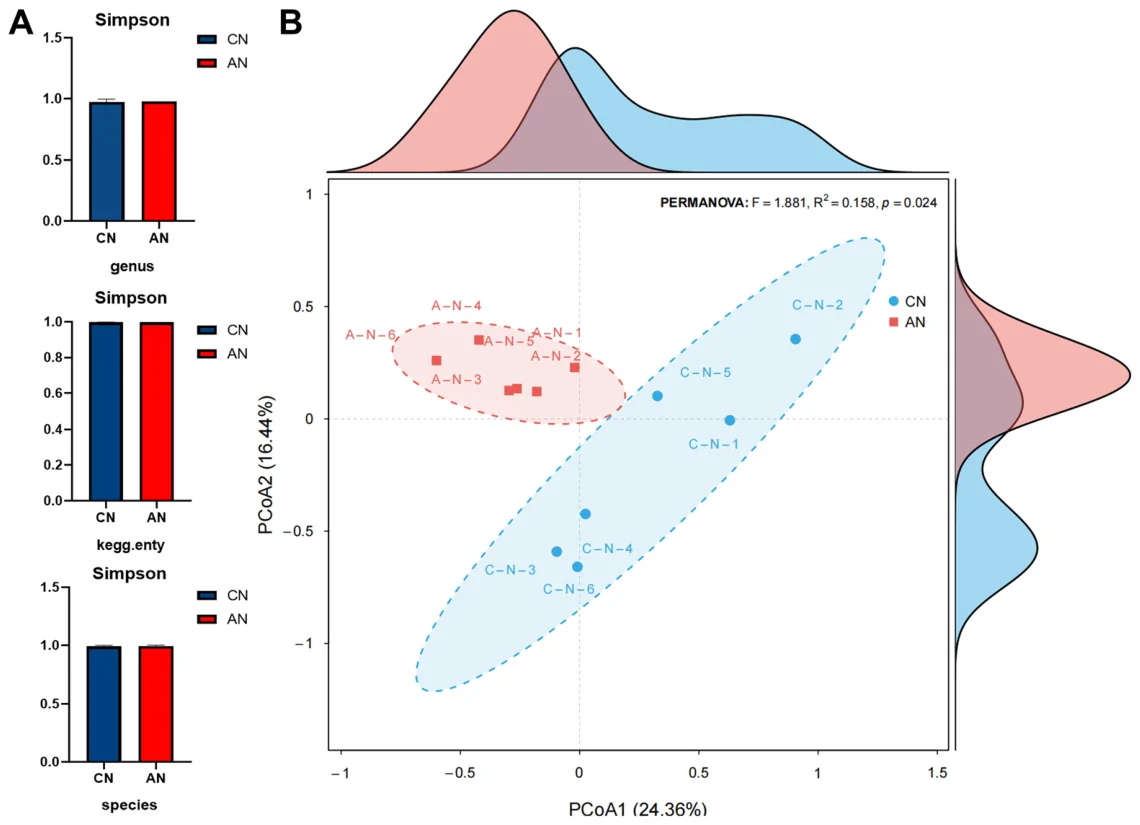
Colonic microbial diversity in weaned rabbits receiving dietary enramycin. (**A**) Simpson index at the genus, KEGG-entry, and species levels; bars represent the mean ± standard deviation. (**B**) PCoA based on Bray–Curtis dissimilarities of TPM-normalized gene-abundance profiles, with 95% confidence ellipses and marginal density curves. *n* = 6.

The Simpson index did not differ significantly between the AN and CN groups at the genus, KEGG-entry, or species level (*p* > 0.05; Figure 1A). PCoA1 and PCoA2 explained 24.36% and 16.44% of the total variation, respectively. PERMANOVA identified a significant group difference in the gene-abundance profiles (pseudo-F = 1.881, R^2^ = 0.158, *p* = 0.024). PERMDISP showed no significant difference in multivariate dispersion between groups (F = 1.351, *p* = 0.272; Figure 1B).

#### 3.2.2. Microbial Composition Analysis

**Figure 2:**
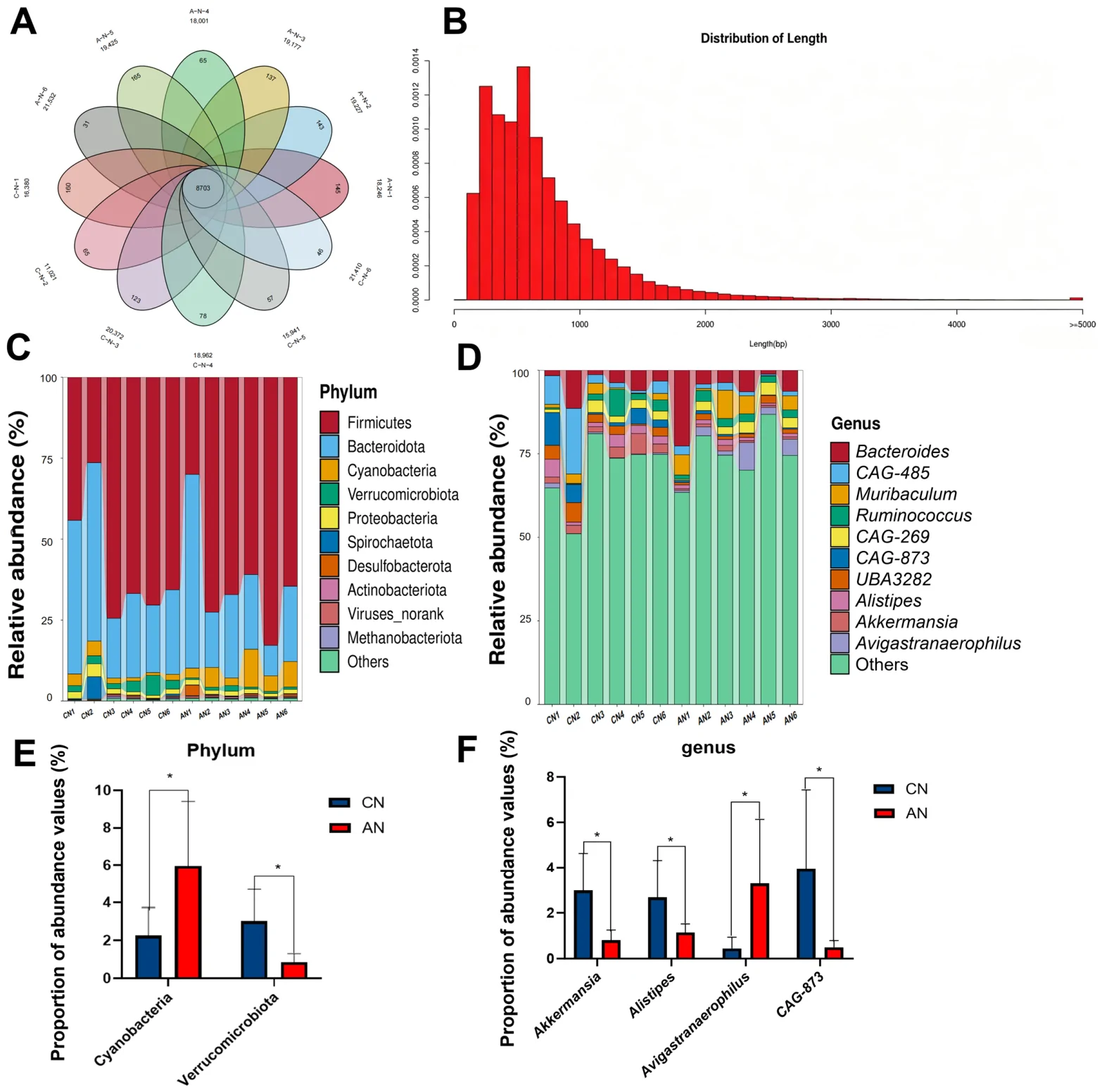
Colonic microbial composition in weaned rabbits receiving dietary enramycin. (**A**) Venn diagram of the samples; (**B**) Length distribution of representative sequences in the non-redundant gene catalog; (**C**) Bar plot of species distribution at the phylum level; (**D**) Bar plot of species distribution at the genus level; (**E**,**F**) Differences in major taxa at the phylum and genus levels. *n* = 6. * indicates significance at *p* < 0.05.

Metagenomic sequencing data were analyzed at both the phylum and genus levels to characterize the effects of enramycin on the composition of the colonic microbiota. A total of 8703 species were shared between the AN and CN groups according to the Venn analysis (Figure 2A). Following construction of a non-redundant gene catalog, the lengths of representative genes were predominantly distributed between 200 and 800 bp, with a peak at approximately 300–400 bp (Figure 2B). Relative abundances were analyzed at the phylum and genus levels to further characterize microbial composition. At the phylum level, Firmicutes and Bacteroidetes were the predominant taxa in both groups (Figure 2C and Table S8). Compared with the CN group, the relative abundance of Cyanobacteria was significantly higher in the AN group (*p* < 0.05), whereas *Verrucomicrobiota* was significantly lower (*p* < 0.05; Figure 2E and Table S8). No marked differences were observed in the dominant phyla between groups.

At the genus level (Figure 2D,F and Table S9), the relative abundances of *Akkermansia*, *Alistipes*, and the Bacteroidales family taxon *CAG-873* were significantly lower in the AN group than in the CN group (*p* < 0.05). In contrast, *Avigastranaerophilus* was significantly enriched following enramycin treatment (*p* < 0.05). Together, these results demonstrate that enramycin treatment was associated with shifts in specific microbial taxa despite the overall stability of dominant community members.

#### 3.2.3. Functional Annotation of the Microbiota

**Figure 3:**
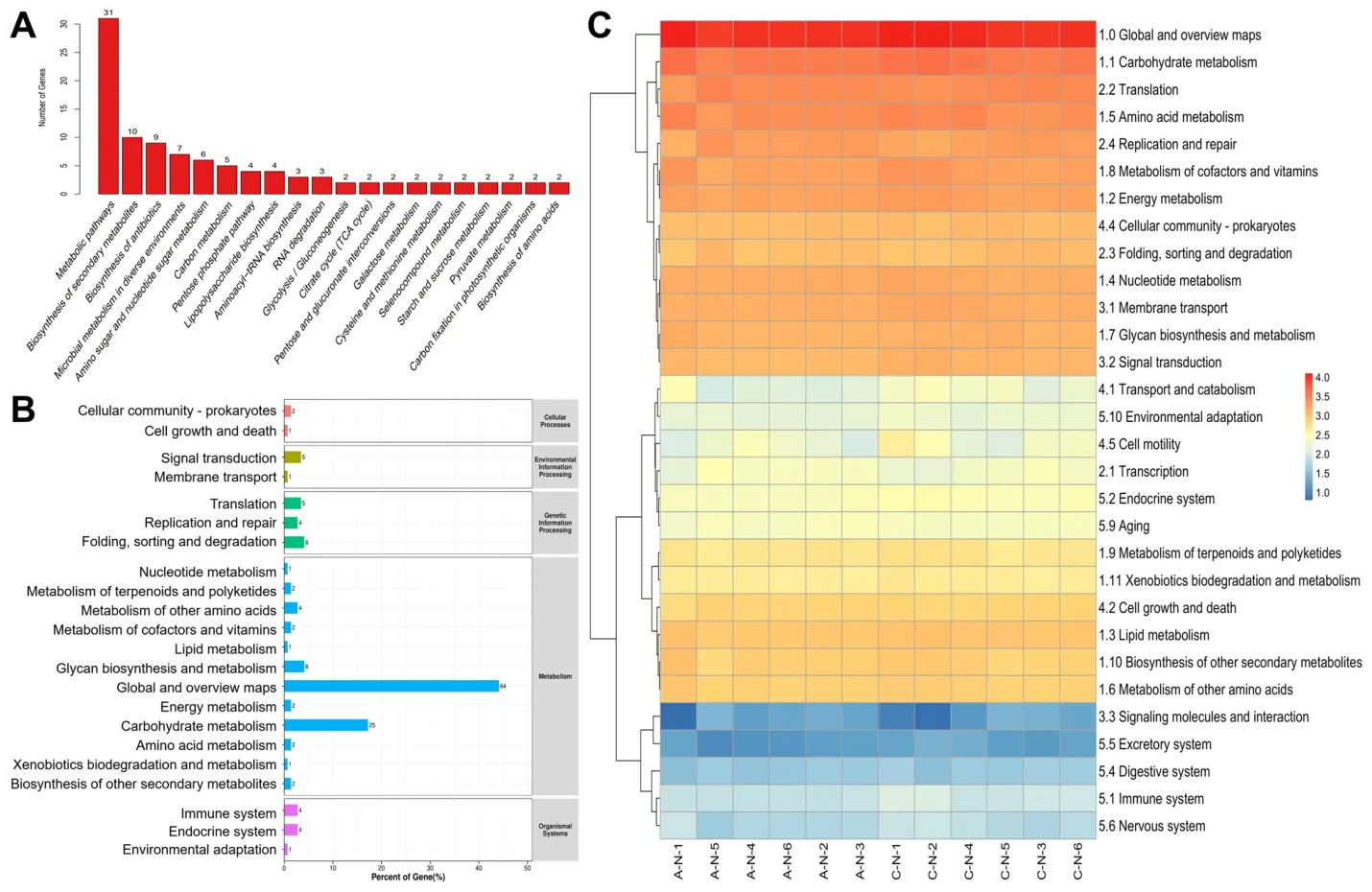
KEGG functional annotation of colonic microbial genes in weaned rabbits. (**A**) Overall KEGG annotation; (**B**) Top 20 functional categories; (**C**) KEGG pathway-class composition. *n* = 6.

KEGG functional annotation was performed to describe the functional composition of all microbial genes annotated to the KEGG database (Figure 3 and Table S10). The largest number of annotated genes was assigned to metabolic pathways, followed by biosynthesis of secondary metabolites, biosynthesis of antibiotics, and microbial metabolism in diverse environments (Figure 3A). In the top 20 KEGG functional categories generated from all annotated genes, global and overview maps accounted for the largest proportion, while carbohydrate metabolism, amino acid metabolism, metabolism of cofactors and vitamins, energy metabolism, and cellular community also showed relatively high gene counts (Figure 3B). The KEGG pathway heatmap based on all annotated genes showed the distribution pattern of functional categories across samples rather than statistically tested differential pathways. In this heatmap, global and overview maps and carbohydrate metabolism were consistently prominent categories, whereas environmental adaptation, signal transduction, excretory system, digestive system, immune system, and nervous system showed lower relative abundance (Figure 3C). Together, these results indicate that the colonic microbial gene set was mainly concentrated in core metabolic functions, providing a functional background for interpreting the observed microbial compositional shifts following enramycin treatment.

**Figure 4:**
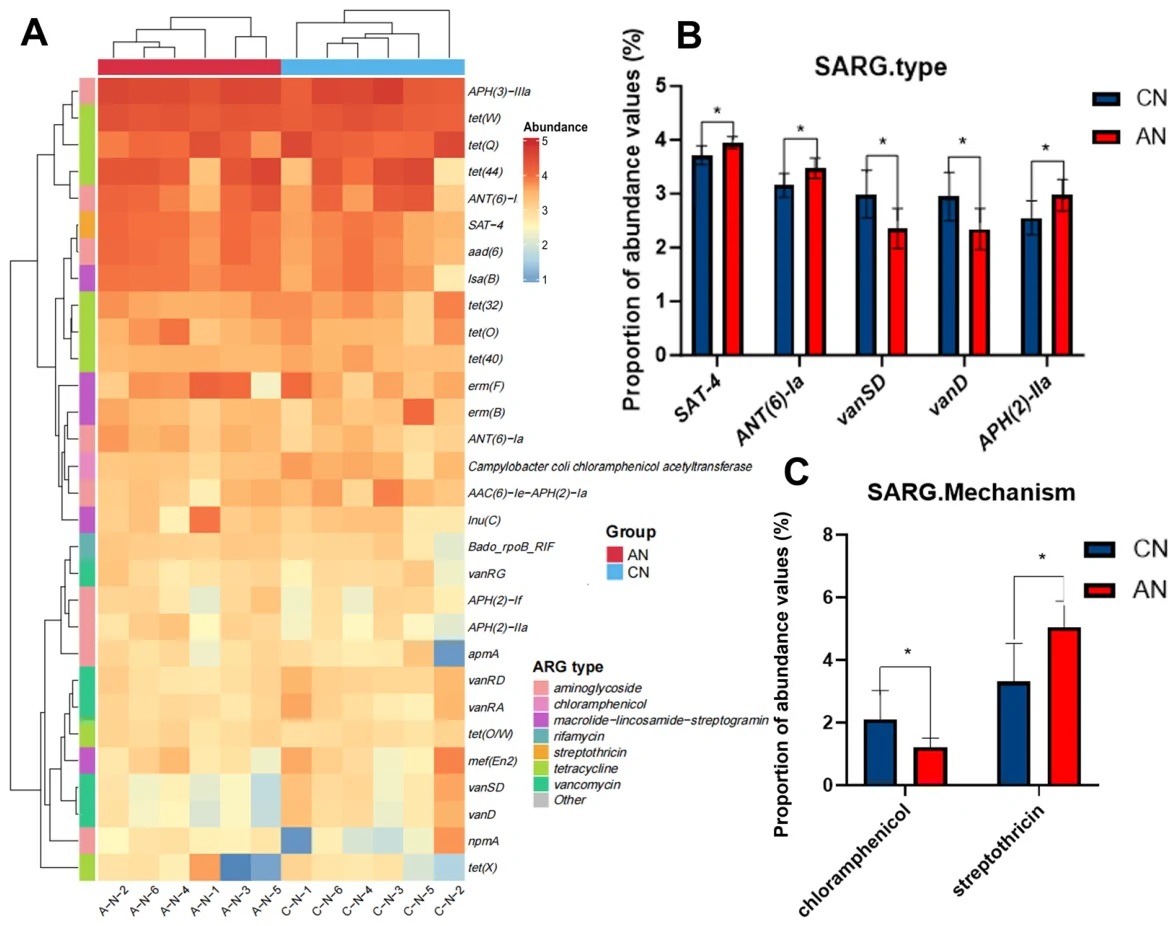
Antibiotic resistance gene profiles in weaned rabbits receiving dietary enramycin. (**A**) Heatmap of resistance gene class abundance; (**B**) selected resistance gene comparisons; (**C**) resistance-mechanism categories. Bars in panels (**B**,**C**) represent the mean ± standard deviation. *n* = 6. * indicates significance at *p* < 0.05.

To further explore the functional potential of the colonic microbiota, antibiotic resistance genes were analyzed (Figure 4A). Five resistance gene subtypes showed statistically significant differences between the CN and AN groups (*p* < 0.05; Figure 4B). The relative abundances of *SAT-4*, *ANT(6)-Ia*, and *APH(2)-IIa* were significantly higher in the AN group than in the CN group, while the relative abundances of *vanSD* and *vanD* were significantly lower in the AN group. At the resistance mechanism level (SARG.Mechanism), two functional categories of resistance genes differed significantly between groups (*p* < 0.05; Figure 4C). The relative abundance of chloramphenicol resistance-related features was significantly lower in the AN group, whereas the relative abundance of streptothricin resistance-related features was significantly higher in the AN group.

### 3.3. Effects of Enramycin on Colonic Metabolism in Weaned Rabbits

#### 3.3.1. Evaluation of Between-Group Metabolomic Patterns

**Figure 5:**
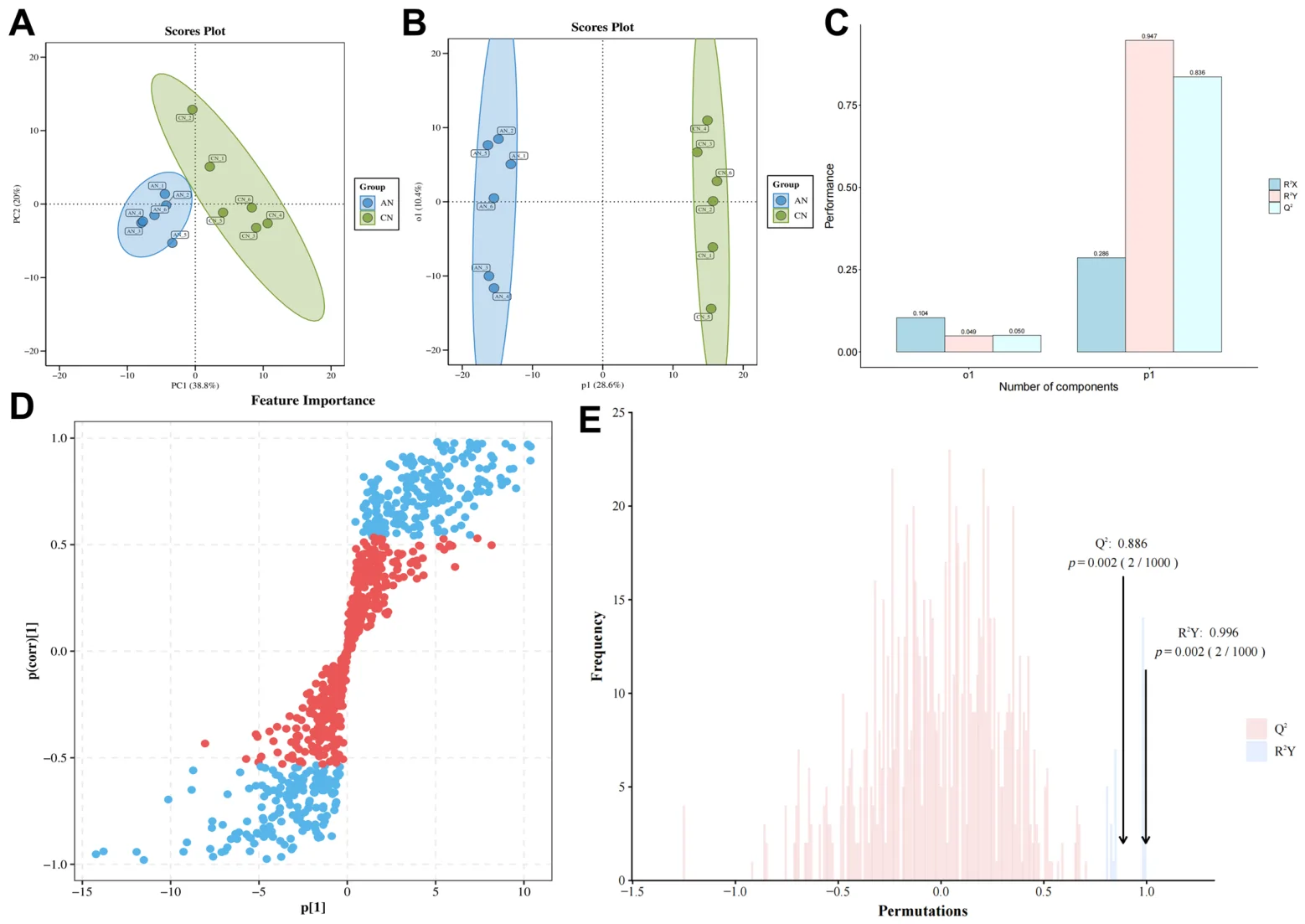
Colonic metabolomic profile analysis in enramycin-supplemented weaned rabbits. (**A**) PCA score plot; (**B**) OPLS-DA score plot; (**C**) OPLS-DA model parameters; (**D**) S-plot, where blue points indicate features with |p(corr)[1]| > 0.5 and red points indicate features with |p(corr)[1]| ≤ 0.5; (**E**) OPLS-DA permutation test plot. *n* = 6.

PCA showed that PC1 and PC2 explained 38.8% and 26.9% of the variation, respectively, and the two groups displayed a separation trend with some within-group variation (Figure 5A). OPLS-DA showed clear separation between the AN and CN groups (Figure 5B). The predictive component had R^2^X = 0.286, R^2^Y = 0.947, and Q^2^ = 0.836 (Figure 5C). S-plot analysis and permutation testing further showed significant differences in the overall metabolite structure between the two groups, with good within-group reproducibility. In the 1000-permutation test, the original R^2^Y and Q^2^ values were 0.996 and 0.886, respectively, and both permutation-test *p* values were 0.002 (Figure 5D,E). These results indicate that enramycin altered the metabolic features of colonic contents in weaned rabbits.

#### 3.3.2. Screening of Differential Metabolites

**Figure 6:**
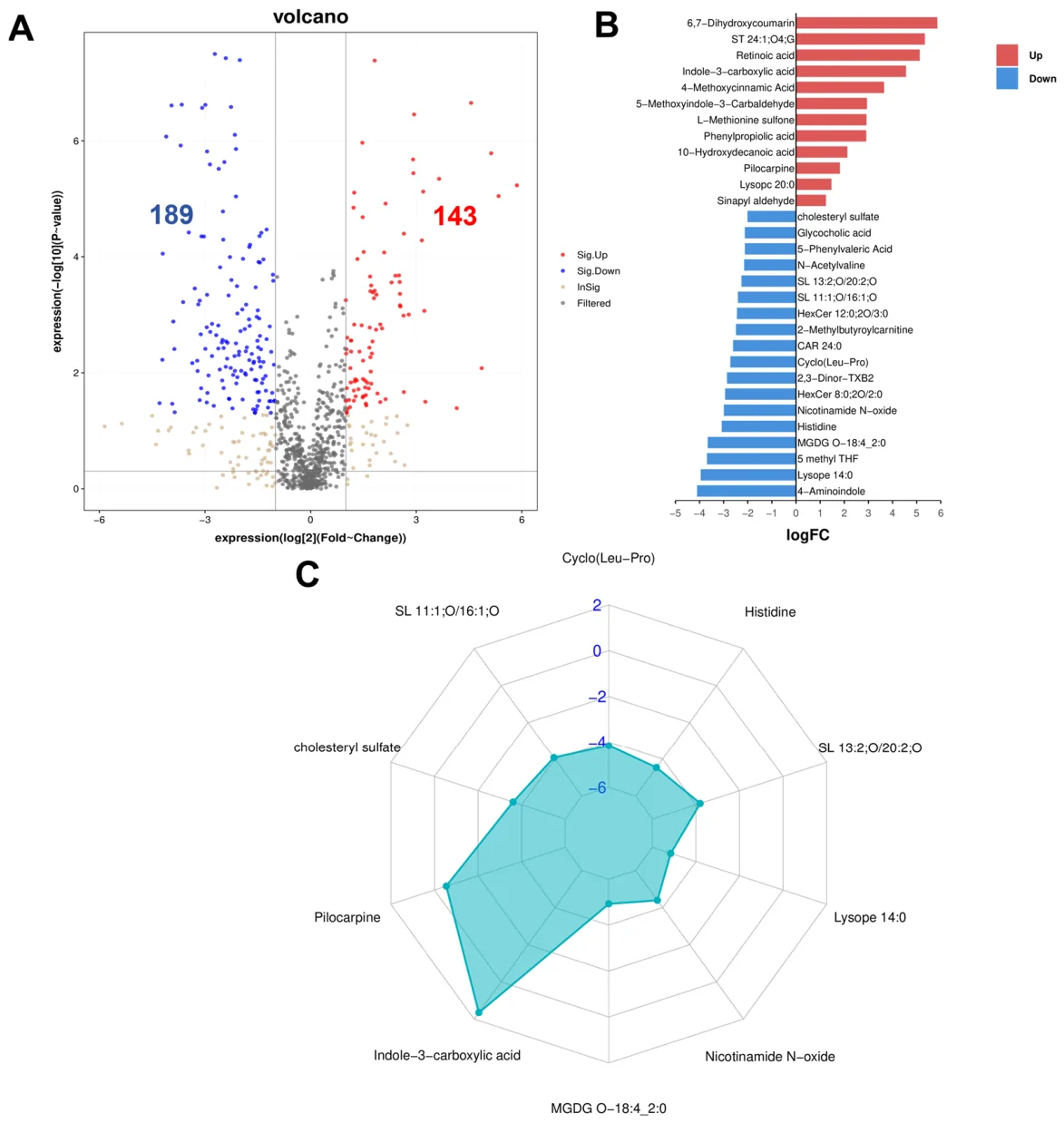
Differential colonic metabolite features in weaned rabbits receiving dietary enramycin. (**A**) Volcano plot; (**B**) Bidirectional bar plot of the top 30 metabolites; (**C**) Relative abundances of selected annotated metabolites. The shaded area is a visual fill representing the radar-profile shape and does not indicate a confidence interval. *n* = 6.

The volcano plot identified 332 differential features in the AN group relative to the CN group, including 143 increased and 189 decreased features (Figure 6A and Table S12). Among these features, 207 had FDR < 0.05. The bidirectional bar plot displayed the top 30 differential features, including 12 increased and 18 decreased features (Figure 6B). Putative annotations showed higher relative abundances of 6,7-dihydroxycoumarin, retinoic acid, indole-3-carboxylic acid, and 5-methoxyindole-3-carbaldehyde, and lower relative abundances of histidine, nicotinamide N-oxide, and MGDG O-18:4_2:0 (Figure 6C).

#### 3.3.3. Pathway Enrichment of Differential Metabolites

**Figure 7:**
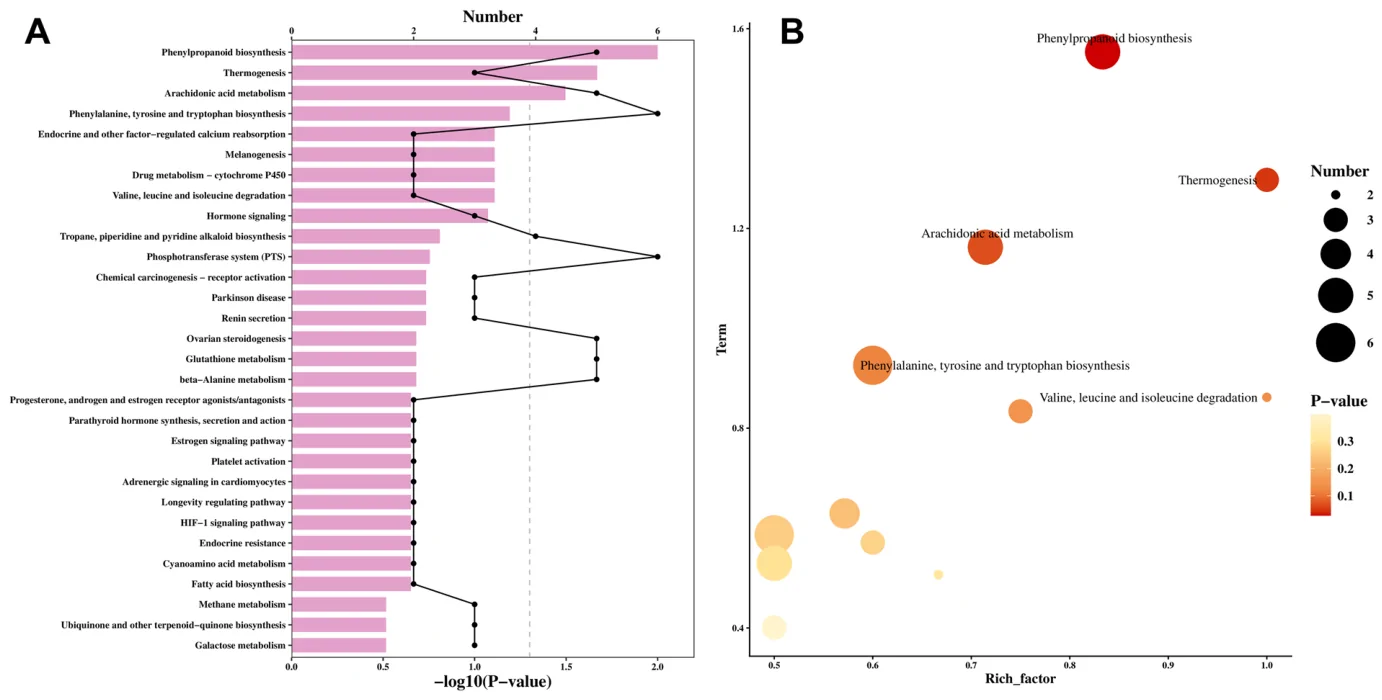
KEGG pathway annotation and enrichment patterns of differential colonic metabolites. (**A**) KEGG enrichment bar plot; (**B**) KEGG enrichment bubble plot.

KEGG pathway analysis identified phenylpropanoid biosynthesis and arachidonic acid metabolism among the prominent annotations of the differential-metabolite set (Figure 7 and Table S13). Furthermore, these differential metabolites exhibited high abundance in the biosynthesis of phenylalanine, tyrosine and tryptophan, as well as in the degradation of valine, leucine and isoleucine; they were also enriched in thermogenesis and calcium absorption processes regulated by endocrine factors. These pathways showed consistent patterns in enrichment factor, gene number, and statistical significance, together forming a metabolic regulatory network. However, no pathway enrichment remained statistically significant after FDR correction.

### 3.4. Integrated Analysis of the Microbiota and Metabolites

**Figure 8:**
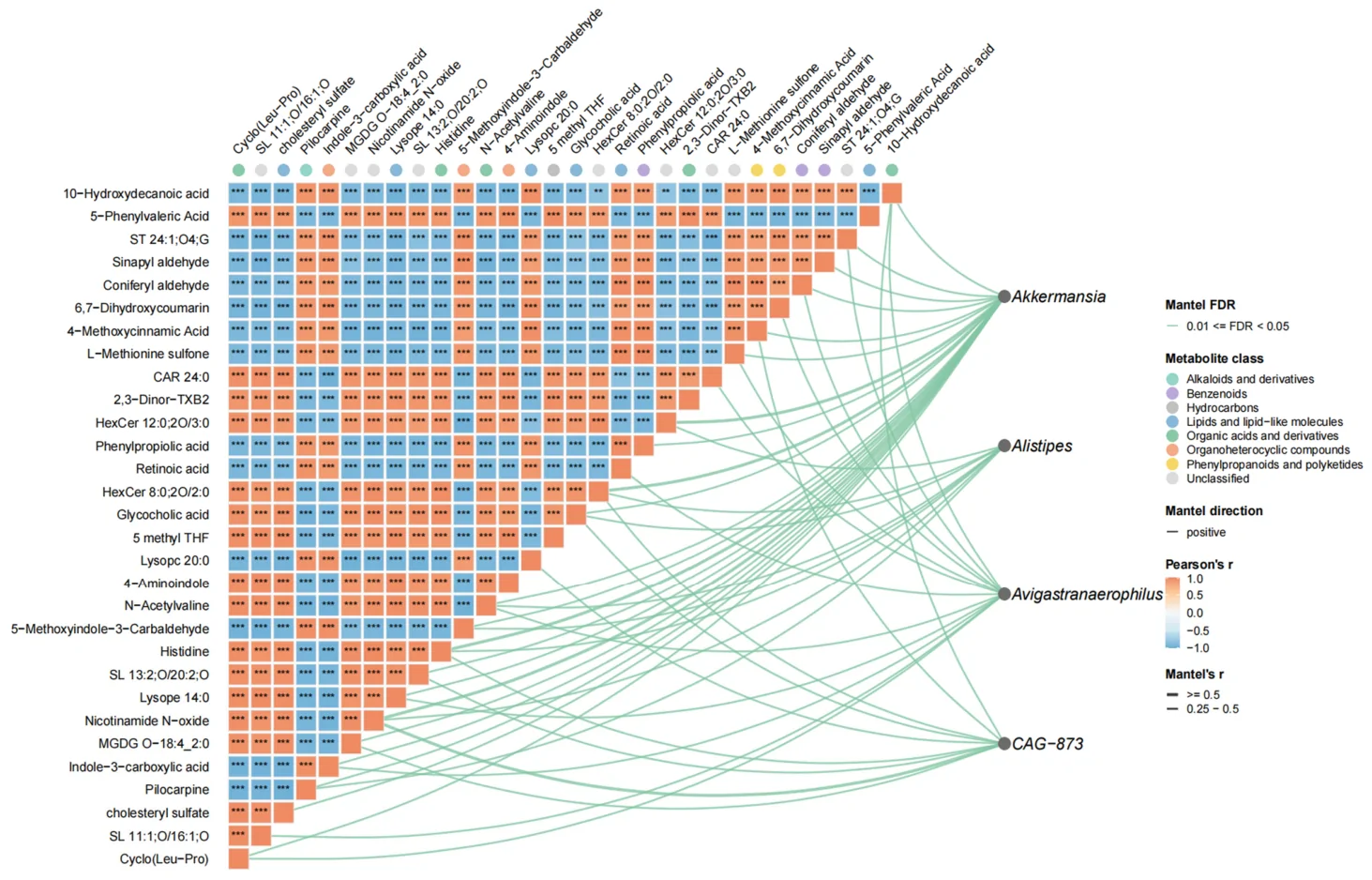
Integrated association analysis of four selected microbial genera and the top 30 differential colonic metabolites. The triangular heatmap shows Pearson correlation coefficients among the metabolites, with orange and blue indicating positive and negative correlations, respectively. Asterisks indicate Benjamini–Hochberg-adjusted significance (** FDR < 0.01, and *** FDR < 0.001), and colored points indicate Class I chemical categories. Curves connect the four genus nodes with metabolite features; orange links indicate FDR < 0.01, green links indicate 0.01 ≤ FDR < 0.05, and link width represents Mantel’s r. All retained Mantel links shown in the network were positive. *n* = 6.

To further explore associations between microbial and metabolic alterations following enramycin treatment, four differentially abundant genera, *Akkermansia, Alistipes*, *Avigastranaerophilus*, and *CAG-873*, were integrated with the top 30 most significant differential metabolites for correlation analysis (Figure 8; Tables S12 and S14). Linked features included retinoic acid, 6,7-dihydroxycoumarin, indole-3-carboxylic acid, 5-methoxyindole-3-carbaldehyde, and several lipid- and organic acid-related metabolites.

The triangular heatmap revealed positive and negative covariation patterns among the top 30 differential metabolites (Figure 8). 10-Hydroxydecanoic acid, 5-phenylvaleric acid, sinapyl aldehyde, coniferyl aldehyde, 6,7-dihydroxycoumarin, and retinoic acid formed a positively correlated cluster, whereas histidine, nicotinamide N-oxide, and MGDG O-18:4_2:0 showed an opposing pattern.

Integration of microbial and metabolomic data further revealed significant associations between differentially abundant genera and metabolite profiles. *Akkermansia* and *Alistipes* were primarily associated with retinoic acid, 6,7-dihydroxycoumarin, 5-methoxyindole-3-carboxaldehyde, and indole-3-carboxylic acid, whereas *Avigastranaerophilus* showed associations with several lipid- and organic acid-related metabolites. Collectively, these results demonstrate coordinated alterations between microbial community composition and colonic metabolic profiles following enramycin treatment.

## 4. Discussion

This study comprehensively evaluated that dietary enramycin produced a distinct short-term response pattern in weaned rabbits by integrating growth and developmental indices with metagenomic, untargeted metabolomic, resistome, and microbiota–metabolite association analyses. During the 14-day trial, it reduced diarrhea incidence without improving body-weight development or major organ indices. Against this divergence between clinical and production-related outcomes, multi-omics analyses identified concurrent changes in the colonic microbiota, metabolome, and selected resistance gene features. Rather than indicating a general growth-promoting effect, these findings show that the short-term health-related response to enramycin was accompanied by broader reorganization of the colonic ecological environment.

### 4.1. Body-Weight Development and Health-Management Outcomes

The post-weaning period is characterized by immature digestive function, incomplete intestinal barrier development, and a microbial community that is still maturing. These features make weaned rabbits particularly susceptible to dietary and environmental disturbances [4,17,35]. Therefore, the lower diarrhea incidence in the enramycin group indicates a measurable short-term health-related response. However, mortality remained unchanged, and the response did not extend to all measured health outcomes (Table 1).

The absence of improved body-weight development may partly reflect the short observation period and the physiological characteristics of rabbit digestion. Rabbits depend strongly on hindgut fermentation for fiber degradation and nutrient recovery [4,36,37,38]. Therefore, the antimicrobial-induced changes in the hindgut community may affect fermentation and nutrient utilization through several potentially opposing processes. Reduced intestinal disturbance would not necessarily result in immediate weight gain if microbial and metabolic functions were reorganized rather than uniformly enhanced. This separation between diarrhea reduction and body-weight development also illustrates the context-dependent effects of dietary antimicrobials. In weaned piglets, enramycin temporarily reduced diarrhea while promoting growth and altering microbial and metabolic profiles [5]. Differences from the present findings may involve host species, diet, baseline health status, dose, or exposure duration. The lower diarrhea incidence observed here may reflect suppression of susceptible microorganisms or temporary stabilization of intestinal function.

### 4.2. Colonic Microbial and Metabolic Features

The microbial findings provide an ecological context for the divergence between the clinical and production-related outcomes. Enramycin altered overall community structure without reducing alpha diversity, indicating selective reorganization rather than generalized microbial depletion. Changes in *Verrucomicrobiota*, *Akkermansia*, *Alistipes*, Cyanobacteria, and *Avigastranaerophilus* further suggest redistribution of ecological niches within the colonic community (Figure 2 and Tables S8 and S9). The biological implications of this restructuring depend on the functions represented within each taxon. *Akkermansia* has been associated with mucus utilization and mucosal ecological interactions, whereas *Alistipes* may participate in polysaccharide fermentation and host metabolic regulation [39,40,41,42,43,44]. Therefore, their reduction could influence substrate availability and microbial interactions within the colon.

The metabolomic findings extend this interpretation from microbial composition to the colonic biochemical environment. Enramycin exposure was accompanied by changes in retinoic acid-, indole-, histidine-, nicotinamide-, lipid-, and organic acid-related features (Figure 6A and Table S12). Retinoic acid contributes to epithelial and mucosal immune regulation, whereas microbial indole derivatives participate in host microbiota signaling [45,46,47,48,49,50]. These annotations suggest that microbial restructuring occurred together with changes in several biologically relevant metabolic processes (Figure 6B). The simultaneous changes in nutrient-related, lipid-related, and microbial-derived metabolites are more consistent with metabolic reorganization than with uniformly enhanced nutrient utilization. Phenylpropanoid biosynthesis and arachidonic acid metabolism were prominent pathway annotations, but no pathway enrichment remained significant after FDR adjustment (Figure 7 and Table S13). This pattern provides a possible ecological context for the absence of measurable body-weight improvement, but it does not establish that the metabolic changes caused the growth response.

### 4.3. Antibiotic Resistance Gene Features

In addition to alterations in microbial composition and metabolic profiles, enramycin treatment was also associated with changes in selected antibiotic resistance gene features (Figure 4 and Table S11). Enramycin was associated with higher streptothricin- and aminoglycoside-related but lower glycopeptide-related resistance features. These opposing directions indicate selective restructuring of particular resistance gene categories rather than a generalized increase in antimicrobial resistance. The observed patterns may arise through direct selection of resistance determinants or indirectly through changes in their microbial carriers. Because enramycin acts predominantly against Gram-positive bacteria [8,9], treatment may alter the abundance of taxa carrying particular resistance genes. In broilers, enramycin-induced resistome changes were similarly accompanied by changes in bacterial hosts carrying specific resistance genes [16]. A comparable community-carrier effect may have contributed to the present results. However, the relative-abundance data describe resistome composition and do not demonstrate corresponding changes in phenotypic antimicrobial resistance. Further studies incorporating larger cohorts, targeted ARG quantification, and longer observation periods will be necessary to determine whether these changes have meaningful implications for resistance dissemination and antimicrobial stewardship in rabbit production systems.

### 4.4. Microbiota–Metabolite Associations and Application Implications

The integrated analysis connected the taxonomic and metabolic responses observed within the omics subset. *Akkermansia* and *Alistipes* were associated with retinoic acid, indole, phenylpropanoid, and aromatic metabolite-related features. *Avigastranaerophilus* was more closely associated with lipid- and organic acid-related features, whereas *CAG-873* contributed additional associations within the network. These patterns suggest that distinct microbial taxa were linked to different components of the altered colonic metabolic environment.

Considered together, the microbial, metabolic, and resistance gene findings support a connected ecological response to enramycin exposure. Community restructuring may alter substrate utilization and metabolic exchange, while changes in microbial carriers may simultaneously reshape resistance gene profiles. The ecological findings should nevertheless be distinguished from the population-level clinical outcome. Diarrhea incidence was evaluated in all rabbits, whereas omics profiling was performed in a smaller subset of surviving rabbits without recorded diarrhea. The omics results therefore characterize treatment-associated responses in diarrhea-free survivors and cannot explain the lower diarrhea incidence in the full population. The limited omics sample size and analysis of colonic samples alone further restrict the generalizability of individual associations to other rabbits or intestinal compartments [29].

Within these boundaries, this study shows that reduced short-term diarrhea incidence can coexist with unchanged body-weight development and coordinated changes in the colonic ecological environment. This pattern supports evaluating dietary antimicrobials from both clinical and ecological perspectives. Longitudinal and post-withdrawal studies should prioritize validation of the key microbial, metabolic, and resistance gene features identified here.

## 5. Conclusions

During a 14-day trial, dietary enramycin supplementation significantly reduced diarrhea incidence without improving body-weight development or major organ indices in weaned rabbits. Integrated metagenomic and untargeted metabolomic analyses characterized concurrent changes in the colonic microbiota and metabolome, together with changes in selected resistance gene features and microbiota–metabolite associations. These findings show that a short-term health-related response can coexist with broader changes in the colonic ecological environment. Given the limited omics sample size and the absence of cecal, post-withdrawal, and functional-validation data, the persistence and biological significance of these patterns require confirmation in larger longitudinal studies.

## Figures and Tables

**Table 1:** Effects of dietary enramycin supplementation on diarrhea incidence, mortality, organ weights, organ indices, and body-weight development in weaned rabbits.

Items	CN	AN	Test	*p* Value	RR, AN vs. CN (95% CI)
Diarrhea incidence, cases/total (%)	8/30 (26.67)	2/30 (6.67)	χ^2^ = 4.320	0.038	0.25 (0.062–0.944)
Mortality, cases/total (%)	4/30 (13.33)	2/30 (6.67)	Fisher’s exact	0.671	0.50 (0.111–2.191)
Thymus weight/g	1.17 ± 0.29	1.23 ± 0.52	–	0.815	–
Spleen weight/g	0.49 ± 0.16	0.51 ± 0.12	–	0.760	–
Kidney weight/g	5.73 ± 1.08	6.93 ± 1.23	–	0.104	–
Liver weight/g	25.47 ± 6.41	29.17 ± 7.35	–	0.375	–
Initial body weigh/g	504.50 ± 106.05	514.00 ± 84.90	–	0.867	–
Terminal body weight/g	997.33 ± 246.70	948.17 ± 139.01	–	0.680	–
Thymus index (%)	0.13 ± 0.07	0.13 ± 0.05	–	0.976	–
Spleen index (%)	0.05 ± 0.03	0.06 ± 0.02	–	0.909	–
Kidney index (%)	0.62 ± 0.22	0.73 ± 0.10	–	0.266	–
Liver index (%)	2.76 ± 1.25	3.10 ± 0.75	–	0.580	–

Note: Diarrhea incidence and mortality were calculated from the full experimental population and presented as cases/total (%). Diarrhea incidence was analyzed using Pearson’s chi-square test, whereas mortality was analyzed using a two-sided Fisher’s exact test. Relative risks compare the AN group with the CN group, and 95% confidence intervals were calculated using the Koopman asymptotic score method. Initial body weight, terminal body weight, organ weights and indices were based on the six terminally sampled rabbits per group. Continuous variables were analyzed using independent-samples *t*-tests.

## Data Availability

All metagenomic and metabolomic data generated and analyzed in this study are provided in the Supplementary Materials accompanying this article. The datasets have not been deposited in an external public repository; therefore, no accession numbers are applicable.

## Supplementary Materials

The following supporting information can be downloaded at: https://www.mdpi.com/article/10.3390/ani16172764/s1, Table S1: Statistics on the volume of raw data; Table S2: Statistics on data volume after quality control; Table S3: Proportion of data remaining after removing the host; Table S4: Statistical Table of Sample Assembly Results; Table S5: Statistics on sequence position annotations for sample assembly; Table S6: Statistical table of gene prediction results for the samples; Table S7: Table of gene abundance in various samples; Table S8: Species Distribution at Phylum Level; Table S9: Species Distribution at Genus Level; Table S10: KEGG annotations pathway; Table S11: Classification and Abundance Table of Antibiotic Resistance Genes; Table S12: Total Metabolite Information for Different Combination of Differences; Table S13: KEGG enrichment results for differential metabolites; Table S14: Wilcoxon rank-sum test results for genus-level microbial abundance.
